# Non-thyroidal illness syndrome and isolated low triiodothyronine as prognostic markers in acute illness: a systematic review and meta-analysis

**DOI:** 10.1007/s12020-026-04772-8

**Published:** 2026-09-28

**Authors:** Elisa Gatta, Virginia Maltese, Enrico Papini, Rinaldo Guglielmi, Roberto Negro, Massimo Salvetti, Carlo Cappelli

**Affiliations:** 1 Department of Clinical and Experimental Sciences, SSD Endocrinologia, University of Brescia, ASST Spedali Civili, Brescia, Italy; 2 Centro per la Diagnosi e Cura delle Neoplasie Endocrine e delle Malattie della Tiroide, University of Brescia, Brescia, Italy; 3 Department of Endocrinology and Metabolism, Regina Apostolorum Hospital- Lifenet Health Group, Rome, Italy; 4 Department of Experimental Medicine, University of Salento, Lecce, Italy; 5 Division of Endocrinology. V. Fazzi Hospital, Lecce, Italy; 6 Department of Clinical and Experimental Sciences, Internal and Emergency Medicine, University of Brescia, ASST Spedali Civili di Brescia, Brescia, Italy

**Keywords:** Non-thyroidal illness syndrome, Low T3 syndrome, acute illness, prognosis

## Abstract

**Purpose:**

To evaluate the association between thyroid hormone alterations and mortality in critically ill and cardiovascular patients, and to compare the prognostic performance of non-thyroidal illness syndrome and isolated low triiodothyronine.

**Methods:**

PubMed/MEDLINE, Scopus, and Web of Science were systematically searched for studies published between 2014 and 2026 evaluating thyroid dysfunction and mortality in acute or critical illness. Studies assessing non-thyroidal illness syndrome or isolated low triiodothyronine were included. Random-effects meta-analyses were performed using odds ratios (ORs) with 95% confidence intervals (CIs).

**Results:**

Twenty-two studies were included. Fifteen studies evaluating non-thyroidal illness syndrome (5,230 patients) showed a significant association with mortality (OR 3.19, 95% CI 2.10–4.85), although heterogeneity was substantial (I²=67.5%, p<0.0001). Nine studies evaluating isolated low triiodothyronine (6,282 patients) also demonstrated a significant association with mortality (OR 2.93, 95% CI: 2.04–4.20), with substantial heterogeneity (I²=63.8%, p = 0.0048). Subgroup comparison and exploratory meta-regression showed no significant differences in effect size between the two definitions of thyroid dysfunction.

**Conclusions:**

Both non-thyroidal illness syndrome and isolated low triiodothyronine are associated with increased mortality in critically ill and cardiovascular patients. Although current aggregate data do not demonstrate a clear quantitative superiority of one definition over the other, non-thyroidal illness syndrome may provide a more integrated biological framework for interpreting thyroid dysfunction during acute illness.

**Supplementary Information:**

The online version contains supplementary material available at https://doi.org/10.1007/s12020-026-04772-8.

## Introduction

Thyroid hormones play a central role in cardiovascular physiology, regulating myocardial contractility, vascular resistance, and systemic hemodynamics through both genomic and non-genomic mechanisms. Triiodothyronine (T3), the biologically active form of thyroid hormone, modulates the expression of key cardiac proteins involved in calcium handling and myocardial performance, including sarcoplasmic reticulum Ca²⁺-ATPase and phospholamban, thereby exerting direct effects on cardiac function and vascular tone [1].

In the setting of acute or chronic systemic illness, alterations in thyroid hormone metabolism are frequently observed, leading to the condition known as non-thyroidal illness syndrome (NTIS), or euthyroid sick syndrome. NTIS is characterized by reduced circulating T3 levels, increased reverse T3, and, in more severe cases, decreased thyroxine (T4) and thyroid-stimulating hormone (TSH), in the absence of intrinsic thyroid disease [2, 3]. This syndrome is highly prevalent among critically ill patients, with reported rates up to 70–80% in intensive care unit (ICU) populations [4], and has been described across a wide range of clinical conditions, including sepsis, trauma, burns, and cardiovascular diseases [5].

Traditionally considered an adaptive response aimed at reducing energy expenditure during systemic stress, NTIS has increasingly been associated with disease severity and adverse outcomes. Several observational studies have demonstrated that NTIS is associated with increased mortality, prolonged ICU stay, and higher rates of complications in critically ill patients [6, 7]. In cardiovascular settings, including acute heart failure, low T3 states have also been consistently associated with worse clinical outcomes and increased mortality.

However, the prognostic role of thyroid hormone alterations in critical illness remains incompletely defined. A major limitation of the existing literature is the heterogeneity in the definition and assessment of thyroid dysfunction. In particular, many studies have focused on isolated reductions in circulating T3 levels, without considering the full biochemical pattern that characterizes NTIS. As a consequence, the terms “low T3” and “NTIS” are often used interchangeably, despite reflecting distinct physiological conditions [8].

This distinction may be clinically relevant. While isolated low T3 may represent a sensitive but nonspecific marker influenced by timing of measurement, nutritional status, and acute hemodynamic changes, NTIS reflects a more integrated alteration of the hypothalamic–pituitary–thyroid axis and peripheral hormone metabolism. Therefore, NTIS may reflect a more integrated alteration of systemic endocrine regulation compared with isolated T3 reduction.

To date, no study has systematically compared the prognostic impact of isolated low T3 and fully defined NTIS across clinical settings. Clarifying this distinction is essential not only for improving risk stratification in critically ill patients, but also for interpreting the growing body of literature on thyroid function abnormalities in acute disease.

Therefore, the aim of this systematic review and meta-analysis was to evaluate the association between thyroid hormone alterations and clinical outcomes, and to compare the prognostic performance of isolated low T3 and non-thyroidal illness syndrome through a structured synthesis of the available evidence.

## Materials and methods

### Search strategy and inclusion criteria

A comprehensive literature search of the PubMed/MEDLINE, Scopus, and Web of Science databases was conducted to identify relevant studies evaluating the association between thyroid hormone alterations and clinical outcomes in critically ill and cardiovascular patients.

A review question was defined based on the Population, Intervention, Comparator, Outcome (PICO) framework: What is the association between thyroid hormone alterations (intervention), including non-thyroidal illness syndrome and low T3 levels, and mortality or adverse clinical outcomes (outcome) in critically ill patients (population), compared with patients without NTIS or with normal T3 levels (comparator)?

The search strategy combined terms related to non-thyroidal illness syndrome and thyroid hormone levels, including: (“non-thyroidal illness syndrome” OR “euthyroid sick syndrome” OR NTIS OR “low T3” OR “low triiodothyronine” OR “free T3” OR FT3) AND (“critical illness” OR “critically ill” OR ICU OR “intensive care”) AND (mortality OR survival OR prognosis OR outcome).

The search was restricted to studies published from January 1, 2014, to April 30, 2026, to ensure consistency in the definition of non-thyroidal illness syndrome and to reflect contemporary clinical practice. Only articles published in English were considered. Preclinical studies, conference proceedings, reviews, editorials, and case reports were excluded. To minimize the risk of missing relevant studies, the reference lists of all included articles were also screened.

### Eligibility criteria

The eligibility criteria were chosen taking into account the review question. Exclusion criteria for the systematic review (qualitative analysis) were reviews, letters, comments, editorials, and conference abstracts on the topic of interest.

### Study selection

C.C and E.G. independently read the titles and abstracts of the records generated by the search algorithm. They then determined which studies were eligible based on predefined criteria. Agreement between the two reviewers regarding study inclusion/exclusion was substantial, and disagreements were resolved by consensus discussion.

### Reporting

**Fig. 1:**
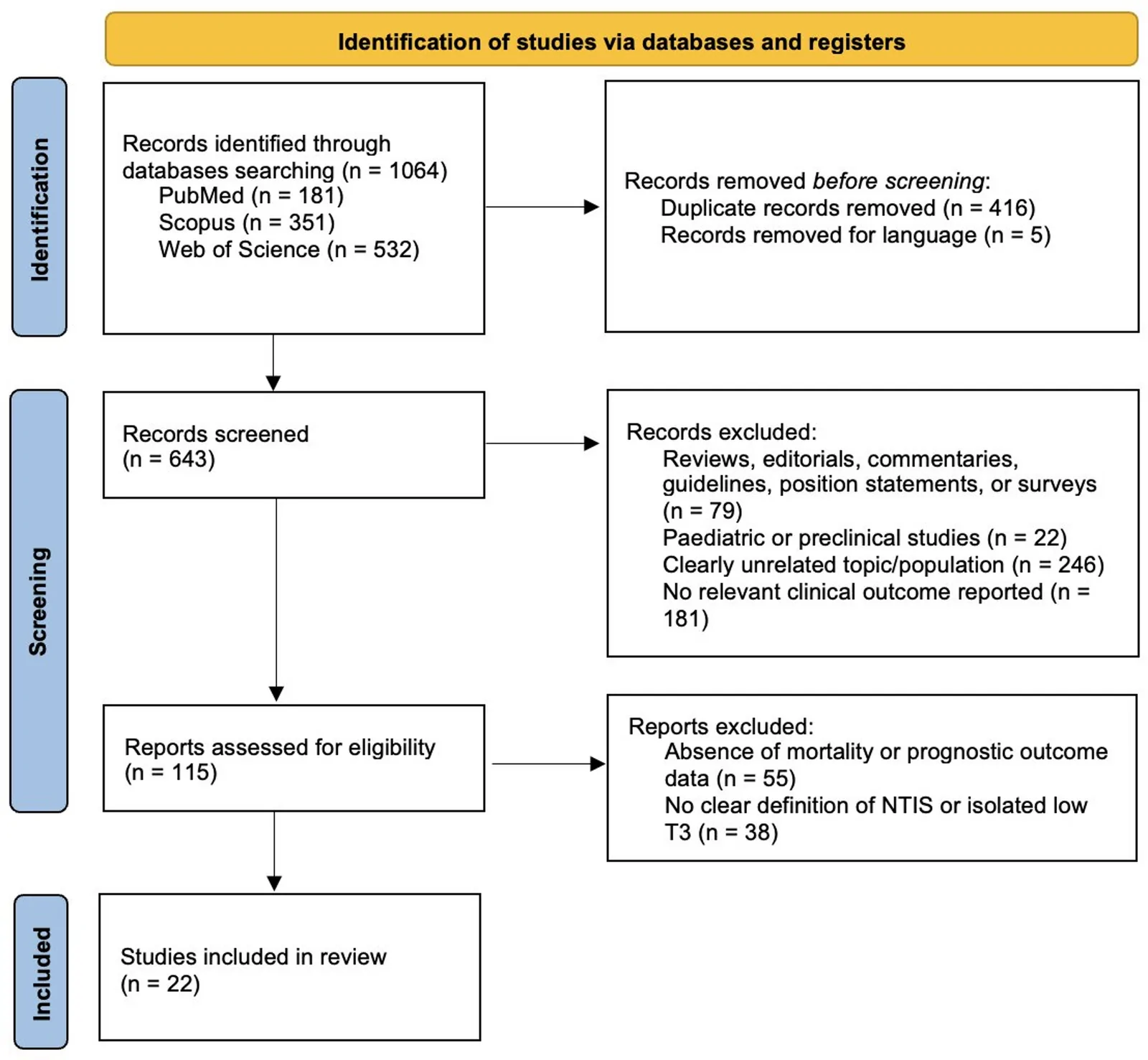
Flowchart of the study selection process for eligible studies

The protocol of this systematic review is registered in PROSPERO (CRD420261391616) and followed the PRISMA (Preferred Reporting Items for Systematic Reviews and Meta-Analyses) guidelines [9]. The corresponding PRISMA flow diagram is reported in Fig. 1. The quality assessment of the studies was carried out using Quality In Prognosis Studies (QUIPS) [10].

### Data extraction

The reviewers independently extracted data from all included studies using full-text articles, tables, and supplementary materials. Extracted data included general study characteristics (authors, publication year, country, study design), patient characteristics (sample size, sex, age, reason for hospitalisation), and thyroid function parameters (T3, FT3, T4, TSH, and, when available, reverse T3), definitions of non-thyroidal illness syndrome and low T3, as well as mortality data. The main findings of the included studies are reported in the Results section.

### Statistical analysis

The data from the included studies were pooled using a random-effects model, accounting for the relative weight of each study. Effect estimates were expressed as odds ratios (OR) with corresponding 95% confidence intervals (CIs) and visually summarized using forest plots. Statistical heterogeneity was assessed using the I² statistic, with values > 50% indicating substantial heterogeneity. A sensitivity analysis was performed using a leave-one-out approach to evaluate the robustness of the pooled estimates. Publication bias was explored through visual inspection of funnel plots and, when appropriate (i.e., ≥ 10 studies), formally assessed using Egger’s regression test [11].

Subgroup analyses were performed according to the definition of thyroid dysfunction (non-thyroidal illness syndrome vs. isolated low T3), with pooled estimates calculated using random-effects models. Differences between subgroups were assessed using the Q-test. An exploratory meta-regression was conducted to evaluate whether the definition of thyroid dysfunction influenced the magnitude of the effect estimates. A mixed-effects model with restricted maximum likelihood (REML) was fitted using the type of thyroid dysfunction as a categorical moderator.

All statistical analyses were performed using R (version 4.5.2) in RStudio (version 2025.05.1 + 513). Random-effects meta-analyses were conducted using the meta package, and exploratory meta-regression was performed using the metafor package.

## Results

### Literature search

A total of 1064 records were identified through database searching. After removal of 416 duplicates and 5 non-English records, 643 records were screened by title and abstract. Of these, 115 reports underwent full-text assessment for eligibility, and 22 studies were ultimately included in the review [12–33] (Fig. 1). In detail, seven studies were prospective in nature; most included studies originated from Asia (*n* = 16), followed by Europe (*n* = 4) and North America (*n* = 2).

Risk of bias was assessed using the QUIPS tool. Outcome measurement and attrition were generally at low risk, whereas study participation, prognostic factor measurement, and confounding were more frequently judged as moderate risk. The main limitations were the retrospective single-center design of several studies, heterogeneous patient populations, variable timing of thyroid hormone assessment, and incomplete adjustment for illness severity. Overall, risk of bias was judged as moderate in most studies, with a few studies at high risk (Supplementary Table S1).

The main characteristics of the studies and their results are briefly presented in Table 1, 2 and 3.

**Table 1:** Characteristics of the studies considered for the review

First author	Ref. *N*.	Year	Country	Study design	*N* Pts.	Sex M/F	Age
*Non thyroidal illness syndrome*							
Liu	[12]	2016	China	Retrospective observational	503	275 / 228	63 ± 18
Padhi	[13]	2018	India	Prospective observational	360	210 / 150	NA
Wang	[14]	2019	China	Prospective cohort	659	412 / 247	64 ± 8
Guo	[15]	2020	China	Prospective observational	305	209 / 96	55 ± 16
Zou	[16]	2020	China	Retrospective cohort	149	71 / 78	47 (36–62)
Deng	[17]	2022	China	Retrospective cohort	186	90 / 96	51 ± 1
Okoye	[18]	2022	Italy	Observational cohort	176	93 / 83	83 ± 7
Swistek	[19]	2022	Poland	Retrospective cohort	215	122 / 93	68 (58–78)
Praveen	[20]	2023	India	Prospective observational	119	84 / 35	60 ± 15
Krug	[21]	2023	Germany	Retrospective cohort	1409	870 / 539	NA
Akbaş	[22]	2024	Turkey	Retrospective cohort	80	46 / 34	72 ± 10
Wang	[23]	2024	China	Retrospective cohort	175	108 / 67	66 ± 16
Elmas	[24]	2025	Turkey	Retrospective cohort	386	205 / 181	65 (36–89)
Li	[25]	2025	China	Prospective observational	545	377 / 168	66 (54–78)
Lebiedzińska	[26]	2026	Poland	Retrospective cohort	157	57 / 100	56 (44–67)
*Low triiodothyronine*							
Rothberger	[27]	2017	United States	Prospective observational	137	76 / 61	75 ± 14
Padhi	[13]	2018	India	Prospective observational	360	210 / 150	NA
Rothberger	[28]	2021	United States	Prospective observational	162	105 / 57	67 ± 17
Asai	[29]	2020	Japan	Retrospective cohort	1190	627 / 563	74 (65–81)
Shigihara	[30]	2021	Japan	Retrospective cohort	2425	1787/638	NA
Zhang	[31]	2022	China	Retrospective cohort	169	76 / 93	74 (64–83)
Krug	[21]	2023	Germany	Retrospective cohort	1506	911 / 595	NA
Zhong	[32]	2023	China	Retrospective cohort	145	69 / 76	50 (39–60)
Yang	[33]	2024	China	Retrospective cohort	495	400 / 95	63 ± 20

Ref.: references; N.: number; Pts.: patients; NA: not available

.

**Table 2:** Results and main findings of the studies about non thyroidal illness syndrome considered for the review

First author	Reason for hospitalisation	Time to mortality assessment (days)	Mortality		Age (years)	
			NTIS	Not NTIS	NTIS	Not NTIS
Akbaş [22]	COPD with acute hypercapnic respiratory failure (ICU)	In-hospital	19 / 51	4 / 29	71 ± 10	71 ± 7
Deng [17]	COVID-19 infection	In-hospital	11 / 59	3 / 127	59 ± 2	47 ± 1
Elmas [24]	ICU patients (respiratory failure, NIV)	In-hospital	36 / 189	18 / 197	65 (63–86)	65 (43–89)
Guo [15]	ICU patients (mixed: sepsis, trauma, surgery, organ failure)	28	23 / 118	12 / 187	58 ± 17	54 ± 15
Krug [21]	ICU patients (mixed, operative + medical)	In-hospital	116 / 284	185 / 1125	59 (44–72)	61 (52–74)
Li [25]	ICU – Chronic critical illness	30	110 / 356	24 / 189	66 (54–78)	66 (52–79)
Lebiedzińska [26]	Aneurysmal subarachnoid hemorrhage	30	22 / 81	10 / 50	57 (48–67)	52 (39–66)
Liu [12]	Community-acquired pneumonia	30	6 / 160	6 / 343	66 ± 19	62 ± 17
Okoye [18]	COVID-19 and non-COVID pneumonia	30	10 / 118	7 / 58	84 ± 7	82 ± 8
Padhi [13]	Sepsis (ICU patients)	28	54 / 73	16 / 119	NA	NA
Praveen [20]	Critically ill patients (ICU, mixed etiologies)	In-hospital	28 / 84	2 / 35	NA	NA
Swistek [19]	COVID-19 infection	In-hospital	28 / 82	15 / 133	73 (66–82)	65 (53–74)
Wang [14]	High-risk patients undergoing CABG	In-hospital	6 / 387	4 / 272	67 ± 8	61 ± 10
Wang [23]	Emergency inpatients (infection, sepsis, organ failure)	In-hospital	31 / 109	7 / 66	70 ± 15	60 ± 17
Zou [16]	COVID-19 infection	NR	0 / 41	1 / 108	58 (50–66)	41 (31–57)

NTIS: Non-thyroidal illness syndrome; COPD: Chronic obstructive pulmonary disease; ICU: Intensive care unit; COVID-19: Coronavirus disease 2019; NIV: Non-invasive ventilation; CABG: Coronary artery bypass grafting

**Table 3:** Results and main findings of the studies about low triiodothyronine considered for the review

First author	Reason for hospitalisation	Time to mortality assessment (days)	Mortality		Age (years)	
			Low T3	Normal T3	Low T3	Normal T3
Asai [29]	Acute heart failure (ICU patients)	In-hospital	57 / 511	18 / 445	75 (67–82)	73 (62–80)
Krug [21]	ICU patients (mixed, operative + medical)	In-hospital	105 / 381	185 / 1125	68 (56–77)	61 (52–74)
Padhi [13]	Sepsis (ICU patients)	28	60 / 168	16 / 119	NA	NA
Rothberger [27]	Acute heart failure	In-hospital	3 / 66	0 / 71	79 ± 13	73 ± 15
Rothberger [28]	ICU patients requiring invasive mechanical ventilation	In-hospital	51 / 98	12 / 64	70 ± 17	63 ± 16
Shigihara [30]	Non-surgical ICU	In-hospital	131 / 759	112 / 1666	74 (65–80)	68 (59–76)
Yang [33]	Severe pulmonary tubercolosis	28	136 / 383	11 / 112	64 ± 20	61 ± 21
Zhang [31]	Sepsis / septic shock	28	35 / 138	9 / 31	75 (63–83)	73 (66–79)
Zhong [32]	COVID-19	30	6 / 50	1 / 95	57 (48–67)	45 (35–54)

T3: triiodothyronine; ICU: Intensive care unit; COVID-19: Coronavirus disease 2019

### Qualitative analysis

The included studies showed substantial heterogeneity in terms of patient populations, clinical settings, timing of hormone assessment, and definitions of thyroid dysfunction. Studies evaluating non-thyroidal illness syndrome (NTIS) were predominantly conducted in critically ill populations, including ICU patients and those with acute systemic illnesses. Wang et al. demonstrated the prognostic relevance of altered thyroid function in critically ill patients [14], while more recent studies consistently showed that low T3/FT3 or NTIS is associated with worse outcomes, although with variable effect sizes [15, 20, 25, 28].

However, the prognostic role of NTIS was not uniform. Krug et al. showed that NTIS lost significance after adjustment for disease severity [21], and Zhang et al. reported no independent association with 28-day mortality in sepsis, suggesting that NTIS may reflect illness severity rather than act as an independent predictor [31].

Studies focusing on isolated low T3/FT3 more frequently involved cardiovascular populations. Asai et al. demonstrated an independent association between reduced FT3 and long-term mortality in acute heart failure [29], while Shigihara et al. confirmed this relationship in a predominantly cardiovascular ICU cohort [30]. These findings are consistent with earlier evidence from Iervasi et al., identifying low T3 syndrome as a strong predictor of mortality in cardiac disease [7].

In infectious and respiratory settings, similar patterns emerged. Liu et al. reported an association between NTIS and mortality in community-acquired pneumonia [12], and Yang et al. showed that low FT3 was associated with both 28-day and 90-day mortality in severe pulmonary tuberculosis [33]. In COVID-19 cohorts, low T3/NTIS was generally associated with increased severity or mortality, although with limited comparability across studies [17–19, 32].

Finally, Lebiedzińska et al. showed that the magnitude of FT3 reduction, rather than a binary NTIS definition, was independently associated with mortality, supporting a continuous rather than categorical interpretation of thyroid dysfunction [26].

### Quantitative analysis

#### Non thyroidal illness syndrome

**Fig. 2:**
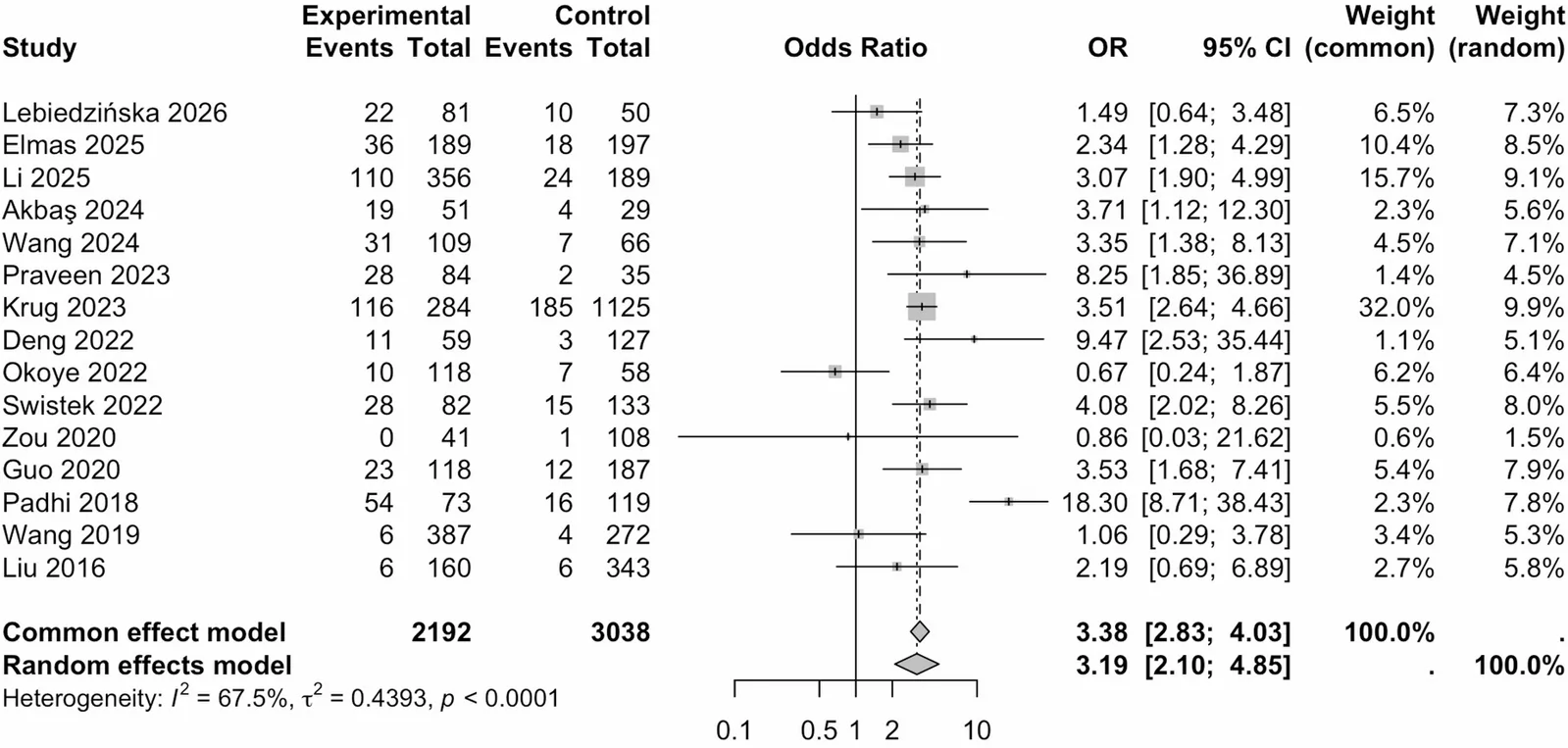
Forest plot of pooled effect estimates comparing mortality risk in patients with non-thyroidal illness syndrome versus controls

A total of 15 studies [12–26], including 5,230 patients (2,192 with NTIS and 3,038 controls), were included in the quantitative synthesis evaluating the association between NTIS and mortality. NTIS was significantly associated with an increased risk of mortality, with a pooled OR of 3.19 (95% CI: 2.10–4.85) using a random-effects model (Fig. 2). Heterogeneity across studies was substantial (I² = 67.5%, τ² = 0.4393; *p* < 0.0001), indicating considerable between-study variability in the magnitude of the effect across clinical settings.

**Fig. 3:**
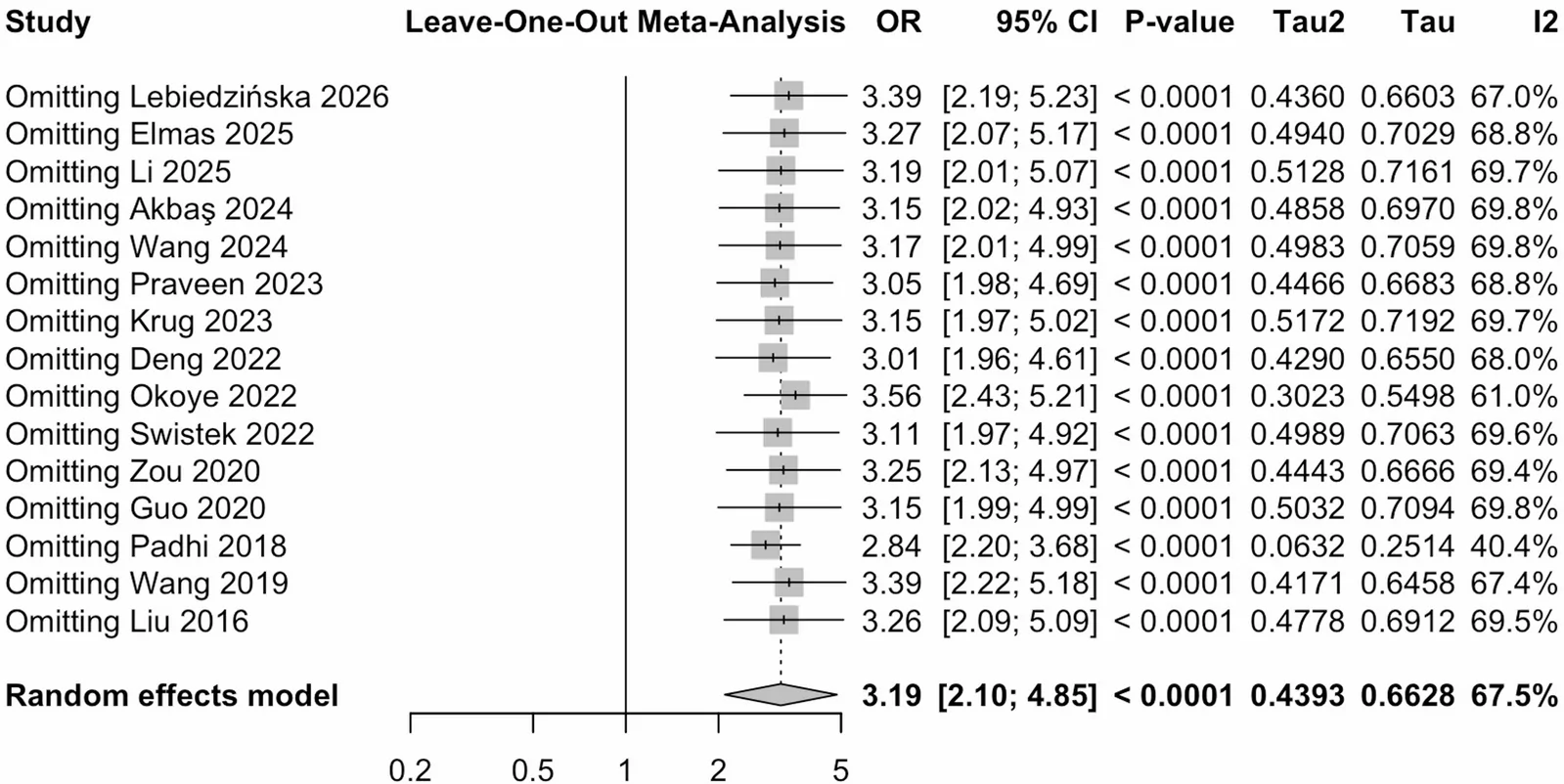
Leave-one-out sensitivity analysis of the pooled odds ratio of the meta-analysis comparing mortality risk in patients with non-thyroidal illness syndrome versus controls

**Fig. 4:**
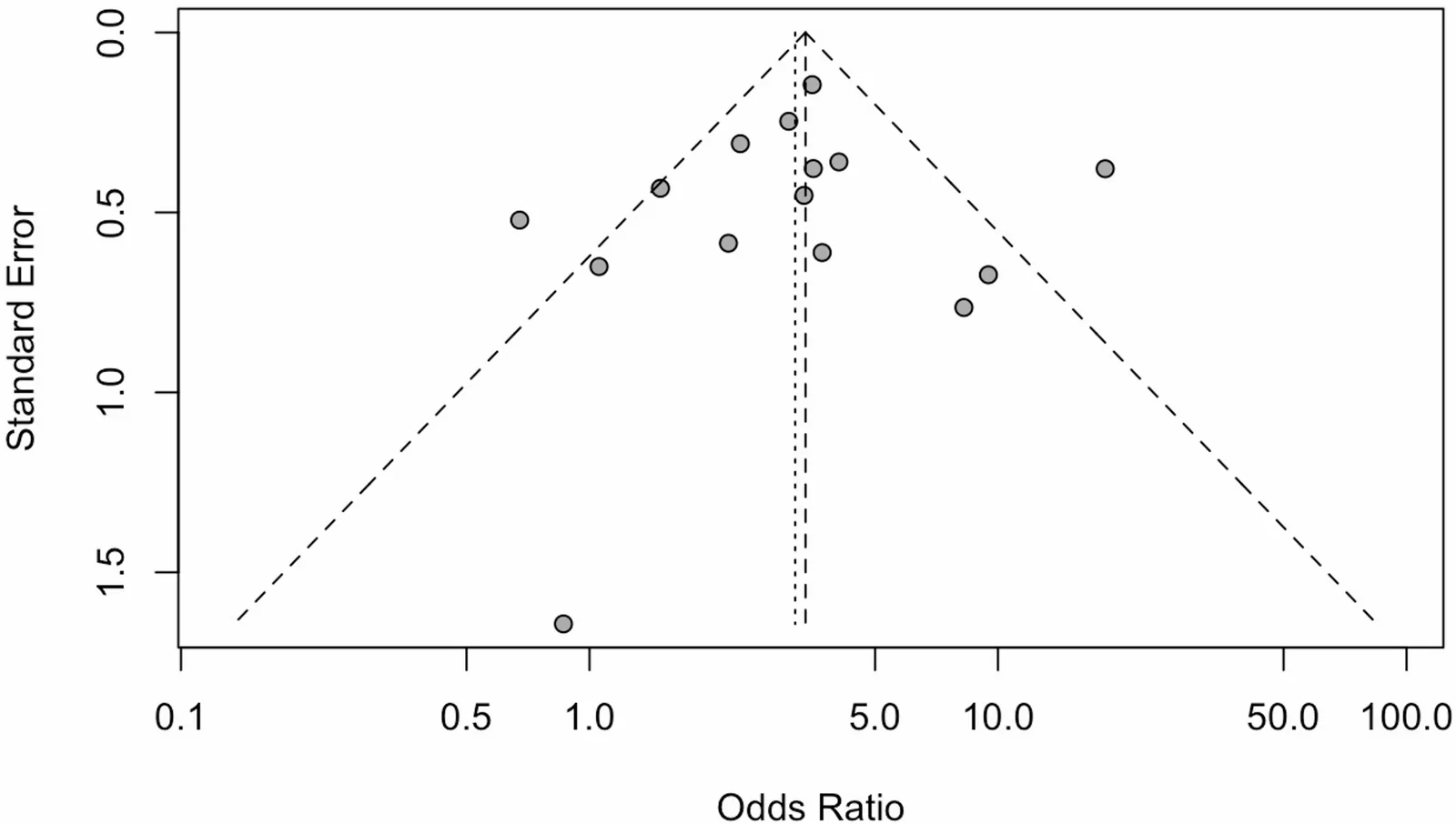
Funnel plot assessing publication bias in the meta-analysis comparing mortality risk in patients with non-thyroidal illness syndrome versus controls

Sensitivity analysis confirmed the stability of the results, with leave-one-out estimates ranging from 2.84 to 3.56, and all remaining statistically significant (*p* < 0.0001). Exclusion of Padhi et al. reduced heterogeneity to 40.4%, suggesting that this study contributed substantially to between-study variability without altering the overall direction or significance of the association (Fig. 3). Visual inspection of the funnel plot did not suggest marked asymmetry (Fig. 4), and Egger’s test did not indicate significant publication bias (*p* = 0.6873).

#### Isolated low triiodothyronine

**Fig. 5:**
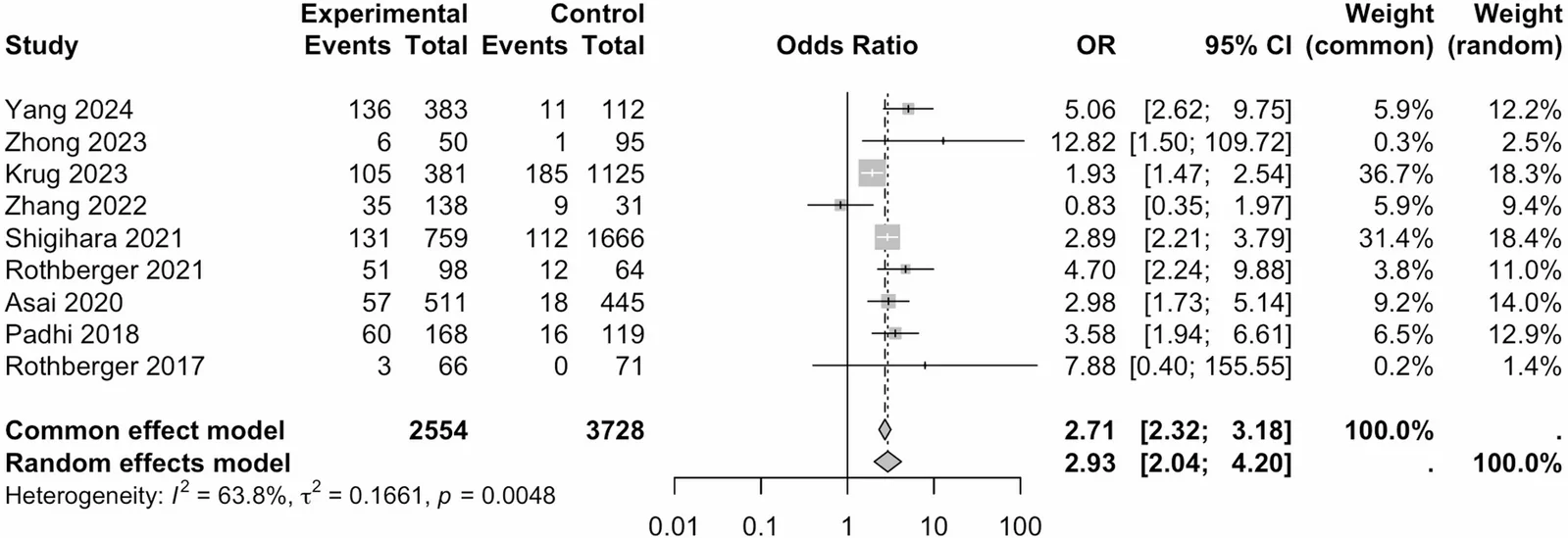
Forest plot of pooled effect estimates comparing mortality risk in patients with low triiodothyronine syndrome versus controls

A total of 9 studies [13, 21, 27–33], including 6,282 patients (2,554 with low T3 and 3,728 controls), were included in the quantitative synthesis evaluating the association between isolated low T3/FT3 levels and mortality. Low T3 was significantly associated with an increased risk of mortality, with a pooled OR of 2.93 (95% CI: 2.04–4.20) using a random-effects model (Fig. 5). Heterogeneity was substantial (I² = 63.8%, τ² = 0.1661; *p* = 0.0048), indicating some between-study variability in the magnitude of the effect across clinical settings.

**Fig. 6:**
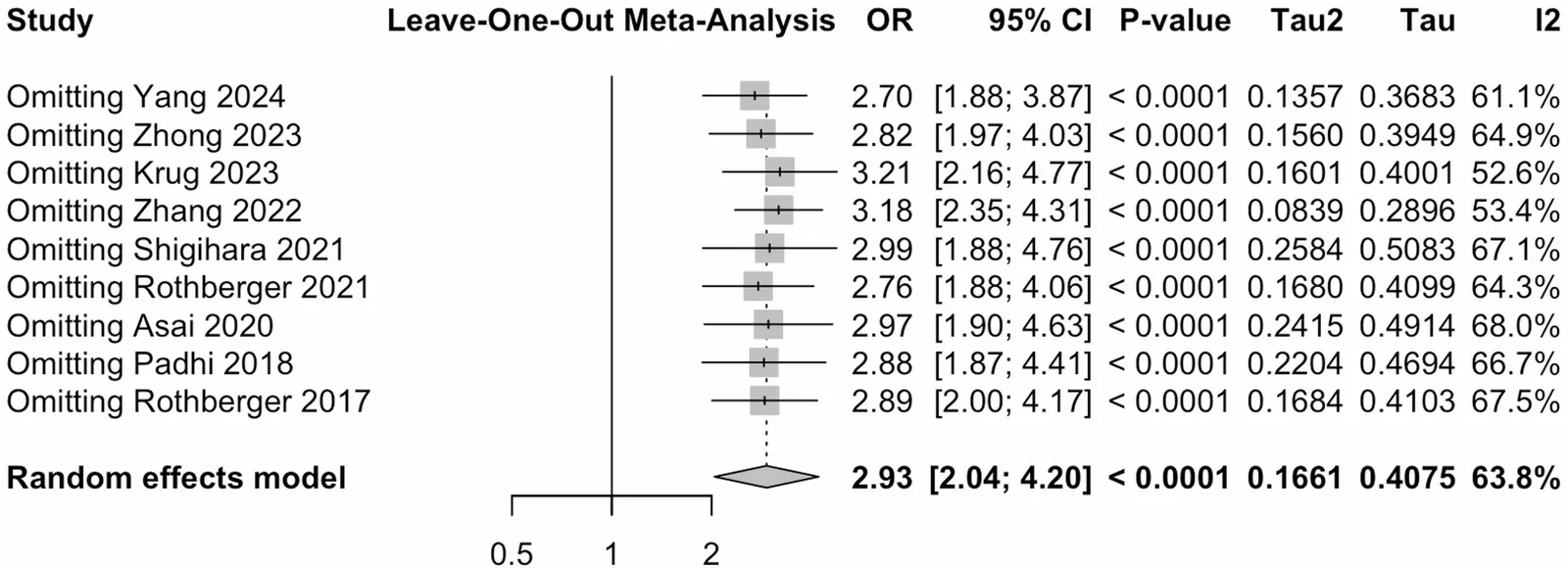
Leave-one-out sensitivity analysis of the pooled odds ratio of the meta-analysis comparing mortality risk in patients with low triiodothyronine syndrome versus controls

**Fig. 7:**
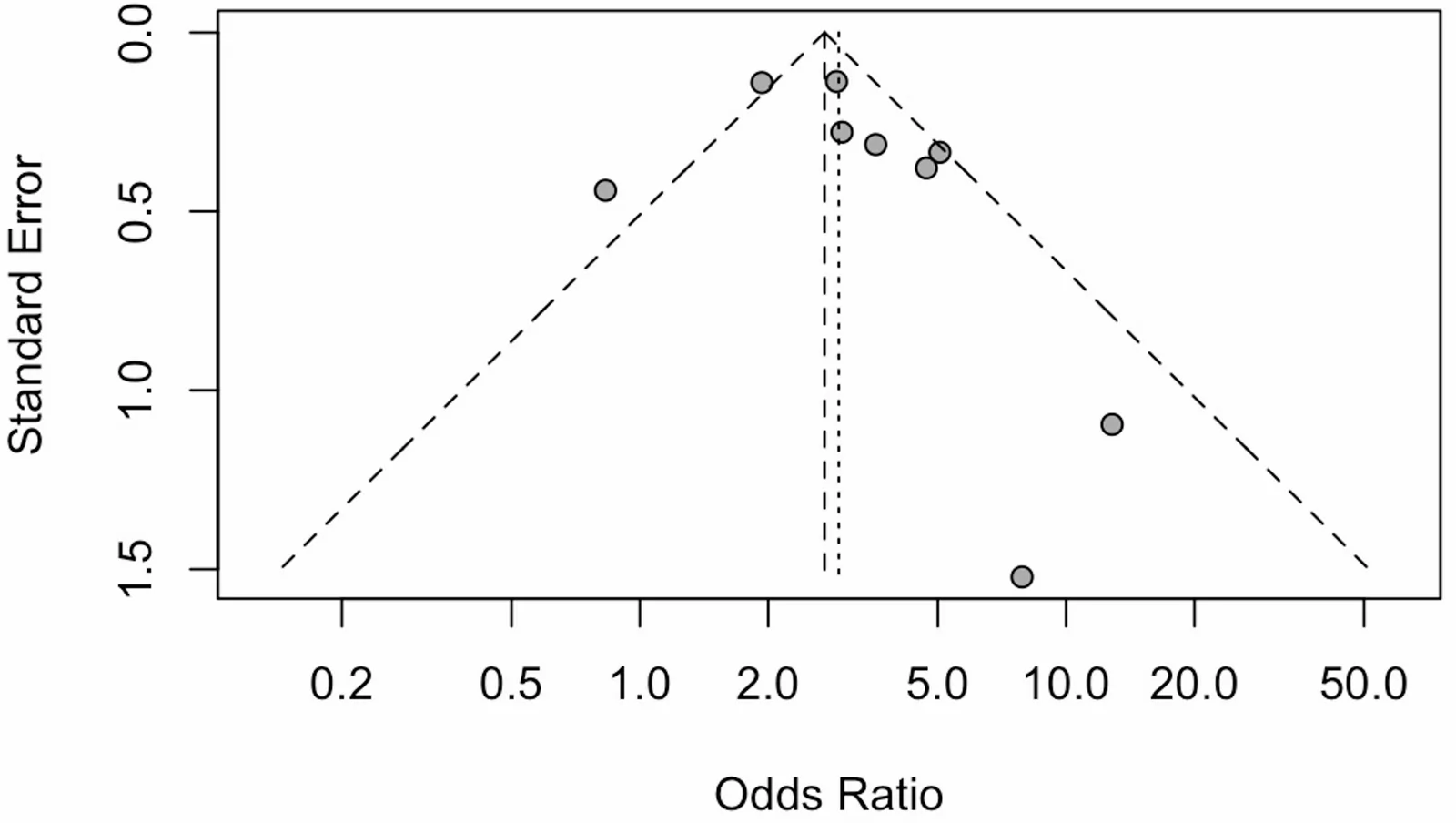
Funnel plot assessing publication bias in the meta-analysis comparing mortality risk in patients with low triiodothyronine syndrome versus controls

Sensitivity analysis confirmed the robustness of the association, with leave-one-out pooled ORs ranging from 2.70 to 3.21, all remaining statistically significant (*p* < 0.0001). Heterogeneity remained moderate to substantial (I² 52.6–68.0%), indicating that no single study was solely responsible for the observed between-study variability. Exclusion of Krug et al. resulted in the largest reduction in heterogeneity (I² from 63.8% to 52.6%), while the pooled estimate remained essentially unchanged (OR 3.21, 95% CI 2.16–4.77) (Fig. 6). Visual inspection of the funnel plot showed some asymmetry with dispersion of smaller studies, although interpretation is limited by the small number of included studies (Fig. 7). Egger’s test was not performed due to the limited number of studies (< 10).

#### Differences between non thyroidal illness syndrome and low triiodothyronine

Direct comparison between the two thyroid dysfunction definitions did not show a statistically significant difference in prognostic effect size. The test for subgroup differences was not significant (*p* = 0.764), and exploratory meta-regression confirmed that the type of thyroid dysfunction definition was not significantly associated with the magnitude of the mortality effect estimate (β = 0.064; *p* = 0.836).

## Discussion

The present systematic review and meta-analysis provide a focused evaluation of the prognostic significance of thyroid hormone alterations in critically ill patients, specifically addressing the distinction between non-thyroidal illness syndrome (NTIS) and isolated low T3. The key finding is that both conditions are associated with increased mortality with broadly comparable effect sizes, and no significant difference between the two definitions was observed in subgroup analysis or exploratory meta-regression. Therefore, the available evidence supports the prognostic relevance of both NTIS and isolated low T3, but does not establish a clear quantitative superiority of one definition over the other.

Despite the absence of a quantitative difference, the two definitions remain conceptually distinct. While isolated low T3 is based on a single biochemical parameter, NTIS represents a broader endocrine phenotype involving coordinated alterations of peripheral thyroid hormone metabolism and hypothalamic–pituitary–thyroid axis regulation [34, 35]. In critical illness, reduced circulating T3 may result from impaired peripheral conversion of T4 to T3, increased reverse T3 production, cytokine-mediated endocrine suppression, and altered TSH regulation [2, 36, 37]. These mechanisms support the interpretation of NTIS as a system-level endocrine response to acute illness rather than a simple isolated hormone reduction. Clinically, this distinction is relevant because isolated low T3 may identify an early or partial biochemical alteration, whereas fully defined NTIS may capture a more advanced disruption of thyroid hormone metabolism and central axis regulation. Nevertheless, the present meta-analysis did not demonstrate a clear quantitative superiority of NTIS over isolated low T3, suggesting that both definitions provide clinically relevant but partly overlapping prognostic information.

Heterogeneity was present in both analyses and likely reflects differences in patient populations, clinical settings, timing of thyroid hormone assessment, assay methods, thresholds used to define thyroid dysfunction, and adjustment for illness severity. These factors limit direct comparison between NTIS and isolated low T3 and support the need for more standardized definitions in future studies. Circulating T3 levels are highly dynamic and influenced by multiple factors, including timing of sampling, nutritional status, pharmacological treatments, and acute hemodynamic changes [37–40]. In addition, the lack of standardized thresholds and assay variability further limit comparability across studies. As a result, low T3 should be interpreted as a context-dependent biomarker rather than a stable clinical construct.

These findings are consistent with previous meta-analytic evidence showing that nonsurvivors in ICU settings have lower levels of circulating thyroid hormones, while TSH levels remain largely unchanged, reflecting a typical NTIS pattern [41]. In this context, our results extend prior evidence by demonstrating that both isolated low T3 and NTIS are associated with increased mortality, while also emphasizing that these two definitions should not be used interchangeably.

Taken together, these observations suggest that the apparent inconsistency of thyroid-related prognostic markers in the literature likely reflects a combination of biological and methodological factors. The key implication of our findings is that the prognostic value of thyroid function in critical illness may lie not in the absolute reduction of a single hormone, but in the disruption of the coordinated endocrine response, which may be better captured when NTIS is rigorously defined as a composite biological phenotype [42].

From a clinical standpoint, NTIS may therefore be better conceptualized as a systems-level marker of disease severity rather than a simple biochemical abnormality [6]. This distinction is relevant for risk stratification, as it supports the interpretation of thyroid function within an integrated framework rather than relying on isolated measurements. At the same time, the absence of a clear superiority in effect size and the observational nature of the available data indicate that NTIS should currently be regarded as a prognostic indicator rather than a therapeutic target.

This study has several strengths, including the explicit differentiation between NTIS and isolated low T3, the use of contemporary studies, and the application of strict definitional criteria, which allowed a structured comparison between two frequently conflated thyroid dysfunction definitions. However, several limitations should be acknowledged. Most included studies were observational and retrospective, limiting causal inference and leaving the possibility of residual confounding, particularly regarding illness severity and comorbidities. In addition, the comparison between NTIS and isolated low T3 remained indirect and should therefore be interpreted cautiously. Two studies [13, 21] contributed data to both the NTIS and isolated low T3 analyses because they reported outcomes according to multiple thyroid dysfunction definitions. Although this approach allowed maximal data extraction, partial overlap between analyses may have introduced a degree of interdependence between subgroups. Furthermore, most studies originated from Asian populations, and five non-English studies were excluded, potentially limiting the generalizability of the findings to other geographic, ethnic, and linguistic settings. Considerable variability in patient age was also observed across studies, with mean ages ranging from approximately 50 to over 80 years, while age was not reported in some cohorts. Finally, several cohorts showed a marked male predominance, particularly in cardiovascular and ICU populations, which may have influenced the observed prognostic associations.

In conclusion, both NTIS and isolated low T3 are associated with increased mortality in critically ill and cardiovascular patients. The similar magnitude of the pooled estimates, together with the absence of significant subgroup or meta-regression differences, suggests that both definitions capture clinically relevant prognostic information. However, NTIS may offer a more integrated biological framework than isolated hormone reduction, because it reflects a broader disruption of thyroid hormone metabolism and hypothalamic–pituitary–thyroid axis regulation. These findings support the need to move beyond single-hormone approaches toward more integrated endocrine phenotyping in critical illness.

## Supplementary Information

Below is the link to the electronic supplementary material.

Supplementary Material 1

## Data Availability

No datasets were generated or analysed during the current study.
