# Supplementary material for: Non-thyroidal illness syndrome and isolated low triiodothyronine as prognostic markers in acute illness: a systematic review and meta-analysis
Source: Endocrine. 2026 Sep 28;91(1):307. doi: 10.1007/s12020-026-04772-8 (PMC13619638; doi:10.1007/s12020-026-04772-8)
Supplement: Supplementary file 1 [file 12020_2026_4772_MOESM1_ESM.pdf]

Supplementary Table S1. Risk of bias assessment according to QUIPS

Study	Study participation	Study attrition	Prognostic factor measurement	Outcome measurement	Confounding	Statistical analysis/reporting	Overall risk
Liu 2016	Moderate	Low	Moderate	Low	Moderate	Low	Moderate
Padhi 2018	Low	Low	Low	Low	Moderate	Low	Moderate
Wang 2019	Moderate	Low	Moderate	Low	Moderate	Moderate	Moderate
Guo 2020	Low	Low	Low	Low	Moderate	Low	Moderate
Zou 2020	Moderate	Low	Moderate	Low	Moderate	Moderate	Moderate
Rothberger 2017	Moderate	Low	Low	Low	Moderate	Moderate	Moderate
Asai 2020	Moderate	Low	Moderate	Low	Moderate	Low	Moderate
Shigihara 2021	Moderate	Low	Moderate	Low	Moderate	Low	Moderate
Rothberger 2021	Moderate	Low	Low	Low	Moderate	Low	Moderate
Deng 2022	Moderate	Low	Moderate	Low	High	Moderate	Moderate
Okoye 2022	High	Low	Low	Low	High	High	High
Swistek 2022	Moderate	Low	Low	Low	Moderate	Low	Moderate
Zhang 2022	Moderate	Low	Low	Low	High	Moderate	Moderate
Praveen 2023	Moderate	Moderate	Moderate	Low	Moderate	Moderate	Moderate
Krug 2023	Moderate	Low	Moderate	Low	Moderate	Low	Moderate
Zhong 2023	Moderate	Low	Low	Low	Moderate	Moderate	Moderate
Akbaş 2024	Moderate	Low	Moderate	Low	Moderate	Moderate	Moderate
Wang 2024	Moderate	Low	Moderate	Moderate	High	High	High
Yang 2024	Moderate	Low	Moderate	Low	High	High	High
Elmas 2025	Moderate	Low	Moderate	Low	Moderate	Moderate	Moderate
Li 2025	Moderate	Low	Low	Low	Moderate	Low	Moderate
Lebiedzińska 2026	Moderate	Low	Low	Low	Moderate	Low	Moderate
